# Programmed cell death in tumor immunity: mechanistic insights and clinical implications

**DOI:** 10.3389/fimmu.2023.1309635

**Published:** 2024-01-12

**Authors:** Man Wang, Fei Yu, Yuan Zhang, Peifeng Li

**Affiliations:** Institute for Translational Medicine, The Affiliated Hospital of Qingdao University, College of Medicine, Qingdao University, Qingdao, China

**Keywords:** programmed cell death, cancer development, tumor immunity, immunosuppression, immunotherapy, anticancer therapeutics

## Abstract

Programmed cell death (PCD) is an evolutionarily conserved mechanism of cell suicide that is controlled by various signaling pathways. PCD plays an important role in a multitude of biological processes, such as cell turnover, development, tissue homeostasis and immunity. Some forms of PCD, including apoptosis, autophagy-dependent cell death, pyroptosis, ferroptosis and necroptosis, contribute to carcinogenesis and cancer development, and thus have attracted increasing attention in the field of oncology. Recently, increasing research-based evidence has demonstrated that PCD acts as a critical modulator of tumor immunity. PCD can affect the function of innate and adaptive immune cells, which leads to distinct immunological consequences, such as the priming of tumor-specific T cells, immunosuppression and immune evasion. Targeting PCD alone or in combination with conventional immunotherapy may provide new options to enhance the clinical efficacy of anticancer therapeutics. In this review, we introduce the characteristics and mechanisms of ubiquitous PCD pathways (e.g., apoptosis, autophagy-dependent cell death, pyroptosis and ferroptosis) and explore the complex interaction between these cell death mechanisms and tumor immunity based on currently available evidence. We also discuss the therapeutic potential of PCD-based approaches by outlining clinical trials targeting PCD in cancer treatment. Elucidating the immune-related effects of PCD on cancer pathogenesis will likely contribute to an improved understanding of oncoimmunology and allow PCD to be exploited for cancer treatment.

## 1 Introduction

Programmed cell death (PCD) is a common process in living organisms that is critical for development, maintenance of cellular homeostasis, immunity and responses to stress (1). PCD is controlled by a wide range of evolutionarily conserved pathways and well-defined mechanisms of action (2). Based on distinct morphological, immunological and genetic features, PCD can be categorized into apoptosis, autophagy-dependent cell death, ferroptosis, necroptosis and pyroptosis (3–5). Conventional cancer treatments mainly rely on cell-suicide programs induced by genotoxic or nongenotoxic stress (6–8). Thus, PCD plays an important role in anticancer therapy-mediated tumor suppression. Moreover, accumulating evidence has demonstrated reciprocal communication between PCD and tumor immunity (9). Based on its ability to trigger an adaptive immune response, PCD can be further grouped into immunogenic and tolerogenic (nonimmunogenic) PCD (10). Immunogenic PCD (e.g., pyroptosis, necroptosis and ferroptosis) reprograms the tumor microenvironment (TME) through extravasation of cellular components, including proinflammatory cytokines and other damage-associated molecular patterns (DAMPs) (9). These signals can be recognized by pattern recognition receptors (PRRs) expressed on innate immune cells, which drives diverse downstream immune responses (11). However, some forms of PCD, such as apoptosis, do not cause the release of cellular contents, and instead culminate in phagocyte-mediated clearance of dead cells without triggering inflammation (12). Immunogenic PCD may finally induce a robust and persistent antitumor immune response, while tolerogenic PCD may facilitate immune tolerance and attenuate the efficacy of anticancer therapy (13). Autophagy is an important cellular mechanism that mediates unnecessary or dysfunctional cytoplasmic constituents to the lysosome for degradation and recycling (14). Autophagy is considered a fundamental prosurvival mechanism that enables cells to avoid excessive PCD. Autophagy can also act to induce cell death, namely, autophagy-dependent cell death, under some circumstances (15). Autophagy-dependent cell death is a type of regulated cell death that mechanistically relies on the molecular machinery of autophagy or its components (15). This form of PCD can be blocked by genetic suppression of at least two components of autophagy pathways (16). The crosstalk between cell death pathways and the TME shapes the complexity and heterogeneity of tumor immunity. Manipulation of PCD is emerging as an attractive approach to improve immunotherapy outcomes (17). Here, we review the molecular mechanisms of common forms of PCD, including apoptosis, autophagy-dependent cell death, pyroptosis and ferroptosis. Furthermore, we summarize the communication among these forms of PCD and the TME and discuss their potential clinical implications in cancer immunotherapy.

## 2 Cell death programs

Based on morphological criteria, cell death can be classified into three subtypes: apoptosis (type I cell death), autophagy-dependent cell death (type II) and necrosis (type III) (18). These cell death processes are executed through different and interlinked signaling cascades that are triggered by distinct stimuli. The term immunological cell death (ICD) was first proposed in 2005 (19). ICD, a form of PCD, is sufficient to activate adaptive immunity (20). Cells undergoing ICD release or expose DAMPs, which promote dendritic cell (DC)-mediated antigen presentation and culminates in the mobilization of cytotoxic T cell responses (21). Various cell death pathways including apoptosis, autophagy-dependent cell death, pyroptosis, ferroptosis and necroptosis have been classified as ICD (22). ICD can be induced by a plethora of therapeutic agents and modalities (23).

### 2.1 Overview of apoptosis

**Figure 1:**
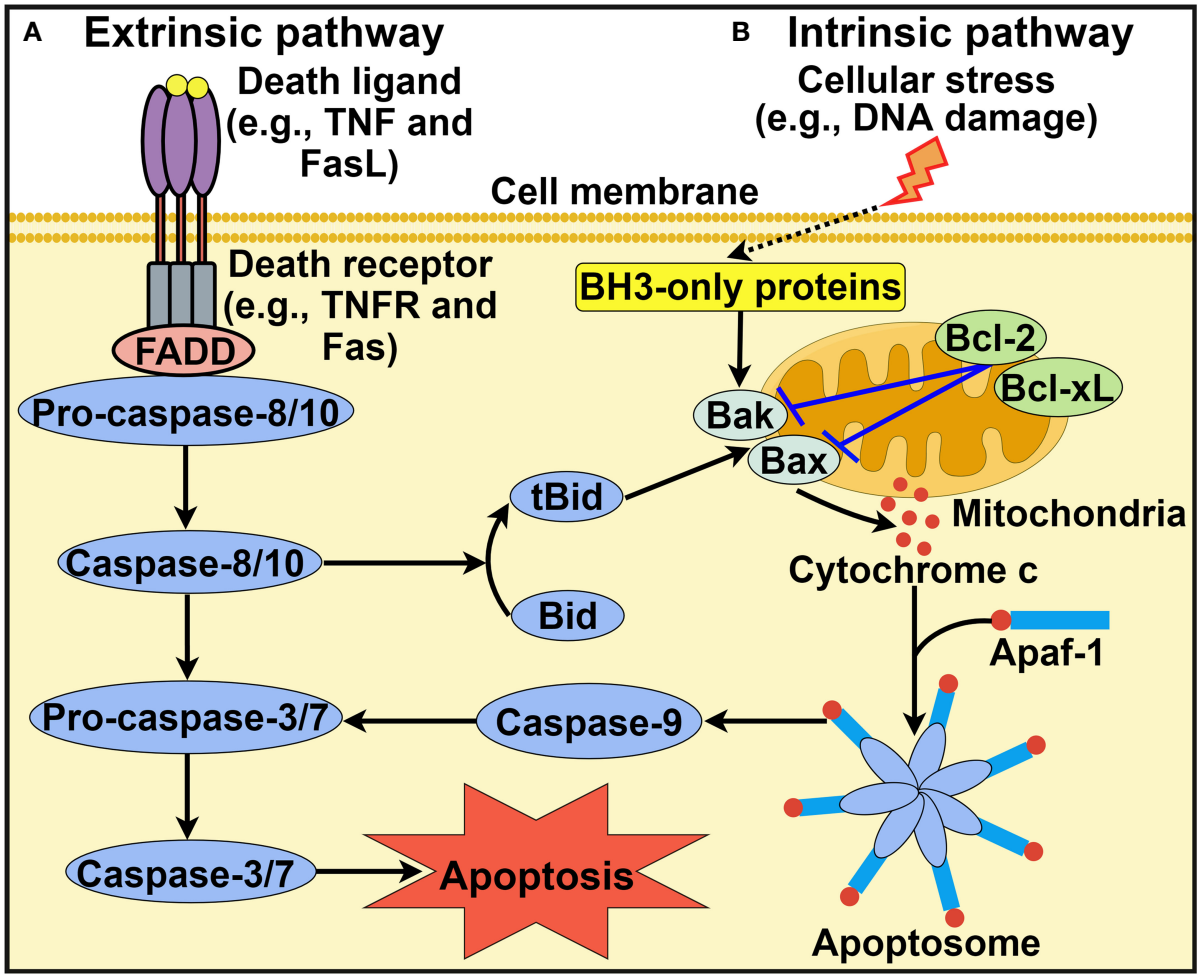
Schematic representation of intrinsic and extrinsic apoptotic pathways. **(A)** Extrinsic apoptotic pathway. Death ligands (e.g., TNF and FasL) bind to their corresponding receptors. This leads to the recruitment of the adaptor protein FADD and pro-caspase-8 or pro-caspase-10. Active caspase-8 or caspase-10 then activates caspase-3 and caspase-7 to driven cell apoptosis. **(B)** Intrinsic apoptotic pathway. This pathway is induced by diverse stress stimuli (e.g., DNA damage and hypoxia) that control the activation of the proapoptotic BH3-only family members Bak and Bax. These two effector proteins cause mitochondrial outer membrane permeabilization, thus favoring the release of cytochrome c into the cytosol. Cytosolic cytochrome c associates with Apaf-1 to form the heptameric apoptosome, which recruits and activates caspase-9. Once activated, caspase-9 cleaves and activates downstream execution caspases (e.g., caspase-3 and caspase-7), culminating in cell apoptosis. In addition, the extrinsic and intrinsic pathways are closely linked. Caspase-8 can convert Bid into its truncated form tBid, which acts to activate Bak and Bax. TNF, tumor necrosis factor; FasL, Fas ligand; TNFR, tumor necrosis factor receptor; FADD, Fas-associated protein with death domain; BH3, B cell lymphoma-2 homology 3; Bcl-2, B cell lymphoma-2; Bcl-xL, B cell lymphoma-extra large; Bak, Bcl-2 antagonist/killer; Bax, Bcl-2-associated X protein; Bid, BH3-interacting domain death agonist; tBid, truncated BH3-interacting domain death agonist; Apaf-1, apoptotic protease activating factor-1. The figure was created by Figdraw (www.figdraw.com).

Apoptosis is the most extensively studied form of PCD and was first described by Kerr et al. in 1972 (24). It is morphologically characterized by cell shrinkage, membrane blebbing, destruction of cell organelles, nuclear and cytoplasmic condensation, DNA fragmentation and rapid phagocytosis of the cell and cell components by neighboring cells (25). Apoptosis relies on a cascade of caspase proteases and is triggered through either extrinsic or intrinsic pathways (26) (**Figure 1**).

#### 2.1.1 Extrinsic apoptotic pathway

The extrinsic apoptotic pathway, also known as the death receptor pathway, is initiated after the association of cell surface death receptors with their corresponding ligands (2) (**Figure 1A**). Members of the tumor necrosis factor (TNF) receptor superfamily, including TNF receptor 1 (TNFR1), CD95/Fas and two TNF-related apoptosis-inducing ligand receptors (TRAIL-R1 and TRAIL-R2) function as death receptors (27). Cytoplasmic death domains of death receptors function as a platform to recruit adaptor proteins such as Fas-associated protein with death domain (FADD) and TNF receptor-associated death domain (TRADD). Adaptor proteins then sequester pro-caspase-8 or pro-caspase-10, which results in the formation of the death-inducing signal complex (DISC) located on the cytoplasmic domain of the ligand-bound death receptor (28). Once the DISC is assembled, caspase-8 or caspase-10 is activated, which cleaves effector caspases (caspase-3 and caspase-7) to induce cell apoptosis. In addition, the FADDosome and ripoptosome, which contain caspase-8, FADD and receptor-interacting protein kinase 1 (RIPK1), are also implicated in the extrinsic pathway. Ataxia-telangiectasia and Rad3-related (ATR)-dependent caspase-10 upregulation promotes formation of the FADDosome (29). Caspase-10 and RIPK1 subsequently recruit TNFR-associated Factor 2 (TRAF2) to the FADDosome, which induces the ubiquitination and degradation of cellular FLICE-inhibitory protein (cFLIP_L_). This leads to nuclear factor-κB (NF-κB) activation that fosters TNF-α production, resulting in caspase-8-mediated apoptosis. In the absence of ATR, caspase-10 and TRAF2, the FADDosome cannot be formed, and caspase-8 cleaves cFLIP_L_ to generate another apoptosis-inducing complex, the FLIPosome. The ripoptosome can change proinflammatory cytokines into death signals, leading to cell apoptosis (30).

#### 2.1.2 Intrinsic apoptotic pathway

Alternatively, the intrinsic pathway of apoptosis, also known as the mitochondrial pathway, is activated by diverse intracellular signals, such as DNA damage, hypoxia, growth factor deprivation and oxidative stress (18) (**Figure 1B**). These stress signals result in mitochondrial outer membrane permeabilization (MOMP) and contribute to the extravasation of pro-apoptotic factors such as cytochrome c from the mitochondria into the cytosol. Members of the B cell lymphoma-2 (Bcl-2) family regulate MOMP and cytochrome c release into the cytoplasm (31). Bcl-2 proteins possess pro- or anti-apoptotic functions. The pro-apoptotic Bcl-2 proteins can be categorized into Bcl-2 homology 3 (BH3)-only activators (Bid, Bim and PUMA), BH3-only sensitizers (Bad, Bik, BMF, HRK and NOXA) and pore-forming effectors (Bcl-2 antagonist/killer (Bak) and Bcl-2-associated X protein (Bax)) (32, 33). Anti-apoptotic Bcl-2 proteins include Bcl-2, B cell lymphoma-extra large (Bcl-xL) and myeloid cell leukemia-1 (Mcl-1). The prevalence of pro-apoptotic Bcl-2 proteins drives apoptotic cell death, while the activation of anti-apoptotic Bcl-2 proteins contributes to the interruption of the apoptotic process (34). The equilibrium between pro- and anti-apoptotic Bcl-2 proteins is crucial for cell fate decisions. Cytosolic cytochrome c can bind to apoptotic protease activating factor-1 (Apaf-1), which leads to the assembly of the apoptosome (35). The apoptosome then recruits initiator pro-caspase-9 via the caspase activation and recruitment domain (CARD), which leads to caspase-9 activation. Caspase-9 then cleaves and activates downstream executioner caspases (e.g., caspase-3 and caspase-7), leading to cell apoptosis. It should be noted that other protein complexes are also involved in intrinsic apoptosis. The PIDDosome is a representative example and is composed of p53-induced protein with a death domain (PIDD), RIP-associated ICH-1/CED-3-homologous protein with a death domain (RAIDD) adaptor and caspase-2 (36). The PIDDosome induces caspase-2 activation in response to DNA damage and genotoxic stress, thus initiating p53-mediated apoptosis.

### 2.2 Overview of pyroptosis

**Figure 2:**
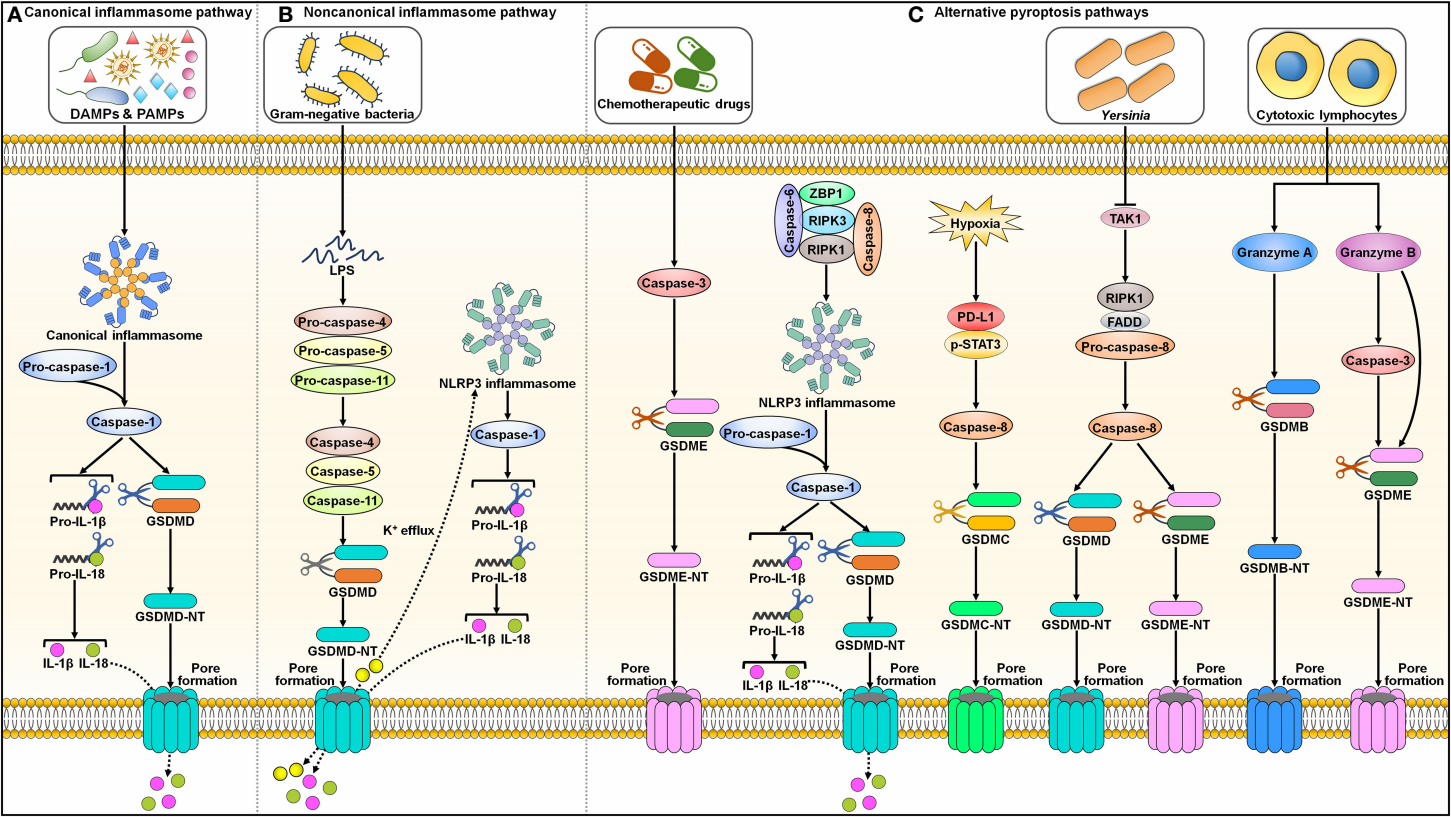
Graphic representation of pyroptotic signaling pathways. **(A)** Canonical inflammasome pathway. Canonical pyroptotic cell death is initiated through the recognition of DAMPs and PAMPs by inflammasome sensors (e.g., NLRP3 and pyrin). This stimulates the assembly of the canonical inflammasome, triggering pro-caspase-1 self-cleavage. Activated capsase-1 cleaves GSDMD to unlash its N-terminal domain (GSDMD-NT). GSDMD-NT binds to the inner leaflet of the cell membrane and oligomerizes to generate membrane pores, leading to the release of cytoplasmic contents including proinflammatory factors. Caspase-1 also processes pro-IL-1β and pro-IL-18 into their active forms (IL-1β and IL-18), which are released through the GSDMD-NT pore. **(B)** Noncanonical inflammasome pathway. The noncanonical pyroptosis pathway is induced by bacterial LPS-mediated activation of human caspase-4/-5 or murine caspase-11. Activated caspase-4, -5 and -11 then cleave GSDMD to facilitate pore formation on the cell membrane. The K^+^ efflux through the GSDMD-NT pore causes the activation of NLRP3 inflammasome and caspase-1. Caspase-1 hydrolyzes pro-IL-1β and pro-IL-18 into biologically active IL-1β and IL-18. These mature cytokines are liberated out of ruptured cells via membrane pores. **(C)** Alternative pyroptosis pathways. Caspase-3 activated by chemotherapeutic drugs shears GSDME to produce a functional GSDME-NT, culminating in pyroptotic cell death. Caspase-6 activates NLRP3 inflammasome to elicit pyroptosis by promoting the interaction between RIPK3 and ZBP1. Under hypoxic condition, p-STAT3 promotes PD-L1 nuclear translocation through coupling with PD-L1. This leads to increased transcription of GSDMC. Following TNF-α treatment, caspase-8 cleaves GSDMC into GSDMC-NT that penetrates the cell membrane to trigger pyroptosis. *Yersinia* motivates RIPK1/caspase-8 signaling cascade through TAK1 blockade. Activated caspase-8 directly processes GSDMD and GSDME, eventually contributing to pyroptosis. In addition, cytotoxic lymphocyte-derived GzmA cleaves GSDMB to actuate pyroptosis. GzmB elicits pyroptotic cell death by both indirectly activating caspase-3 to cleave GSDME and directly shearing GSDME. DAMPs, damage-associated molecular patterns; PAMPs, pathogen-associated molecular patterns; IL-1β, interleukin-1β; IL-18, interleukin-18; GSDMD, gasdermin D; GSDMD-NT, the N-terminal domain of gasdermin D; LPS, lipopolysaccharide; NLRP3, nucleotide-binding oligomerization domain-like receptor family pyrin domain-containing protein 3; GSDME, gasdermin E; GSDME-NT, the N-terminal domain of gasdermin E; ZBP1, Z-DNA binding protein 1; RIPK3, receptor-interacting protein kinase 3; RIPK1, receptor-interacting protein kinase 1; PD-L1, programmed cell death-ligand 1; p-STAT3, phosphorylated signal transducer and activator of transcription 3; GSDMC, gasdermin C; GSDMC-NT, the N-terminal domain of gasdermin C; TAK1, transforming growth factor-β-activated kinase 1; FADD, Fas-associated protein with death domain; GSDMB, gasdermin B; GSDMB-NT, the N-terminal domain of gasdermin B.

Pyroptosis was first described in *Salmonella*-infected macrophages in the 1990s and was initially considered a process of apoptosis (37, 38). Later, it was realized that this bacterium-induced cell death, which mainly relies on caspase-1, was different from the more well-known process of apoptosis. The term pyroptosis was proposed in 2001 to describe this novel type of PCD (39). The major morphological characteristics of pyroptosis include chromatin condensation, DNA fragmentation, cell swelling with large bubbles, cell membrane rupture and the release of intracellular contents (40, 41). Currently, two principal (canonical and noncanonical inflammasome pathways) and several alternative pyroptosis pathways have been identified (**Figure 2**).

#### 2.2.1 Canonical inflammasome pathway

The inflammasome complex typically consists of a sensor (e.g., PRR), the adaptor protein apoptosis-associated speck-like protein containing a CARD (ASC), and an effector protein (e.g., caspase-1) (42). The well-characterized PRRs include absent in melanoma 2 (AIM2), nucleotide-binding oligomerization domain (NOD)-like receptor (NLR) family pyrin domain-containing protein 1 (NLRP1), NLRP3, NLR family CARD-containing protein 4 (NLRC4) and pyrin (43). Inflammasomes formation provides a platform for caspase-1 activation (44) (**Figure 2A**). Activated capsase-1 cleaves gasdermin D (GSDMD) to unlock its N-terminal domain (GSDMD-NT). GSDMD-NT binds to the inner leaflet of the cell membrane and oligomerizes to generate pores that enhance membrane permeability, which results in water influx, cell swelling, cytolysis and extravasation of cytoplasmic contents including proinflammatory factors (45). Caspase-1 also processes the precursor forms of interleukin-1β (IL-1β) and interleukin-18 (IL-18) into their active forms, which are released through the GSDMD-NT pore (46).

#### 2.2.2 Noncanonical inflammasome pathway

The noncanonical pyroptosis pathway is independent of inflammasomes, and instead, this pathway is initiated by bacterial lipopolysaccharide (LPS)-mediated activation of human caspase-4/-5 or murine caspase-11 (47) (**Figure 2B**). Activated caspase-4, -5 and -11 then cleave GSDMD to induce pore formation on the cell membrane (48). Caspase-4, -5 and -11 cannot directly trigger the maturation of pro-IL-1β and pro-IL-18. Potassium (K^+^) efflux through GSDMD-NT pores leads to activation of the NLRP3 inflammasome and caspase-1 (49, 50). Caspase-1 then hydrolyzes pro-IL-1β and pro-IL-18 into biologically active IL-1β and IL-18, respectively. These mature cytokines are liberated from ruptured cells via membrane pores.

#### 2.2.3 Alternative pyroptosis pathways

In addition to caspase-1, other caspases can also induce pyroptosis (**Figure 2C**). Reportedly, caspase-3 activated by chemotherapeutic drugs shears gasdermin E (GSDME) to produce a functional pore-forming fragment (GSDME-NT), culminating in pyroptotic cell death (51, 52). Caspase-6 activates the NLRP3 inflammasome to elicit pyroptosis by promoting the interaction between RIPK3 and Z-DNA binding protein 1 (ZBP1) (53). Programmed cell death-ligand 1 (PD-L1) transforms TNF-α-induced apoptosis into pyroptosis in cancer cells (54). Under hypoxic conditions, phosphorylated signal transducer and activator of transcription 3 (p-STAT3) promotes PD-L1 nuclear translocation through coupling with PD-L1. This leads to increased transcription of gasdermin C (GSDMC). Following TNF-α treatment, caspase-8 cleaves GSDMC into GSDMC-NT, which penetrates the cell membrane to trigger pyroptosis. *Yersinia* activates the RIPK1/caspase-8 signaling cascade through blockade of transforming growth factor-β (TGF-β)-activated kinase 1 (TAK1) (55–57). Activated caspase-8 directly processes GSDMD and GSDME, eventually contributing to pyroptosis.

In addition, granzymes (Gzms) are found to participate in GSDM-dependent pyroptosis. Cytotoxic lymphocyte-derived GzmA cleaves gasdermin B (GSDMB) in target cells, which leads to pyroptosis (58). GzmB elicits pyroptotic cell death in cancer cells by both indirectly activating caspase-3 to cleave GSDME and by directly shearing GSDME (59). The molecular mechanisms of pyroptosis are still poorly understood and warrant in-depth investigation.

### 2.3 Overview of ferroptosis

**Figure 3:**
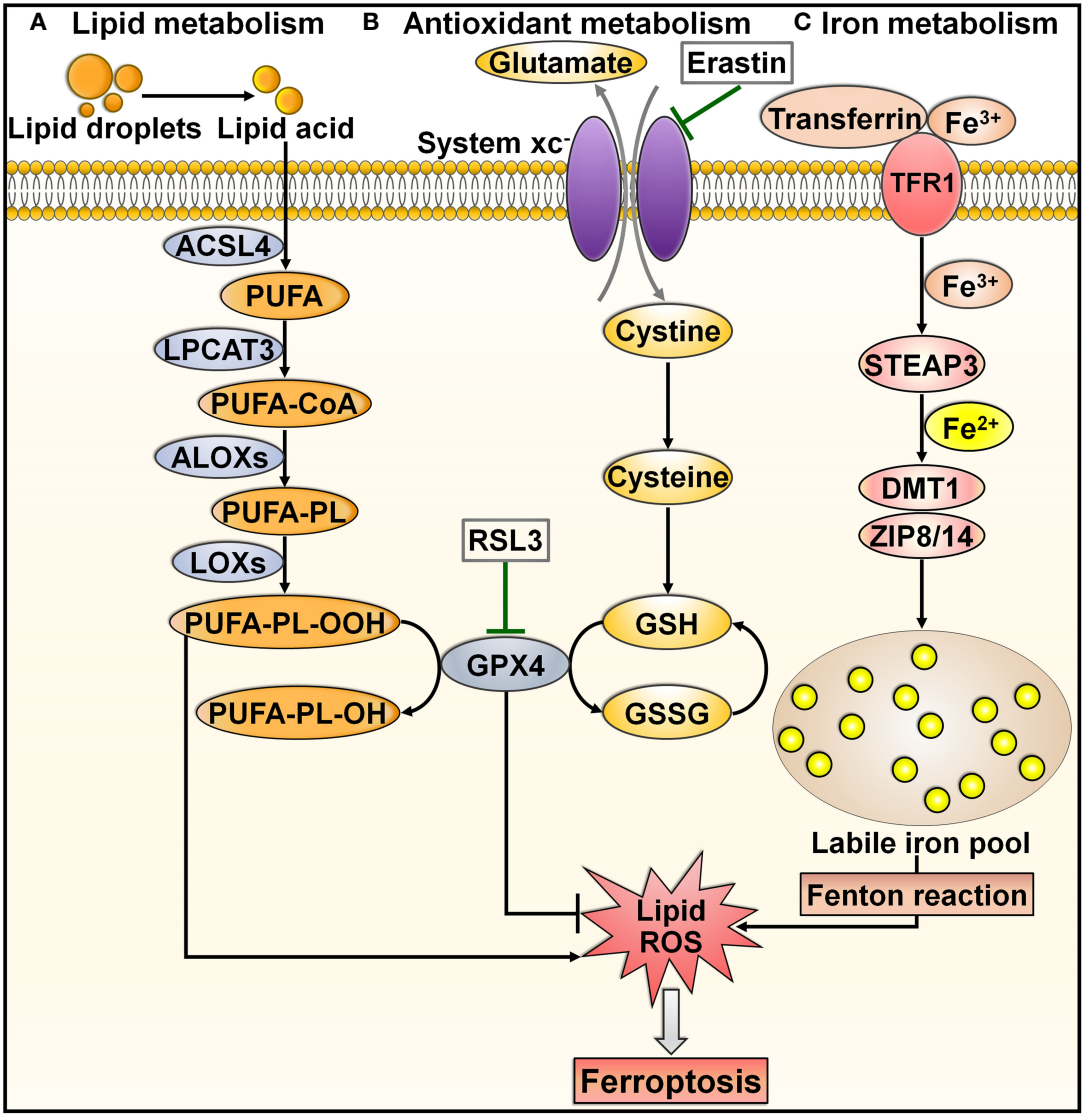
Overview of molecular mechanisms regulating ferroptosis. Ferroptosis is can be induced through either transporter-dependent pathway or enzyme-regulated pathway. **(A)** Lipid metabolism. Under energetic or oxidative stress, free PUFA is catalyzed by ACSL4, LPCAT3 and ALOX to produce PUFA-PL. PUFA-PL is then converted into toxic PUFA-PL-OOH under the assistance of LOXs and eventually induces ferroptotic cell death. **(B)** Antioxidant metabolism. Extracellular cystine is exchanged with glutamate to enter the cell through system xc^-^, and it converts into cysteine to promote GSH synthesis. GPX4 transforms GSH into GSSG and simultaneously reduces toxic lipid hydroperoxides (LOOHs) to nontoxic lipid alcohols (L-OHs), thus protecting cells from ferroptosis. Blockade of system xc^-^ by erastin, or GPX4 inhibitor (e.g., RSL3) can trigger ferroptosis by promoting lipid-ROS production. **(C)** Iron metabolism. Extracellular Fe^3+^ enters the cell through transferrin and TFR1. In cells, Fe^3+^ is released from transferrin and reduced to Fe^2+^ by STEAP3. Fe^2+^ is then transferred into a labile iron pool, which is mediated by DMT1 or ZIP8/14. Excess Fe^2+^ contributes to the formation of massive ROS and consequent oxidative stress through the Fenton reaction. These events drive the peroxidation of lipids, nucleic acids and proteins, culminating in cell ferroptosis. ACSL4, acyl-CoA synthetase long-chain family member 4; PUFA, polyunsaturated fatty acid; LPCAT3, lysophosphatidylcholine acyltransferase 3; PUFA-CoA, coenzyme A-activated polyunsaturated fatty acid; ALOXs, arachidonate lipoxygenases; PUFA-PL, PUFA-containing phospholipid; LOXs, lipoxygenases; PUFA-PL-OOH, PUFA phospholipid hydroperoxide; PUFA-PL-OH, PUFA phospholipid alcohol; RSL3, RAS-selective lethal 3; GPX4, glutathione peroxidase 4; GSH, glutathione; GSSG, glutathione disulfide; ROS, reactive oxygen species; TFR1, transferrin receptor 1; STEAP3, six-transmembrane epithelial antigen of prostate 3; DMT1, divalent metal transporter 1; ZIP8/14, zinc-iron regulatory protein family 8/14.

Ferroptosis is a recently discovered mode of PCD that is mainly characterized by excessive iron accumulation, lipid peroxidation, and cell membrane rupture (60, 61). Ferroptosis inducers were discovered prior to when this process was formally termed. In 2003, Dolma et al. (62) revealed a novel form of nonapoptotic PCD caused by the small molecule erastin. Later, in 2008, synthetic compounds RAS-selective lethal 3 (RSL3) and RSL5 were found to trigger this pattern of cell death (63, 64). It was not until 2012 that such PCD was formally named ferroptosis (65). Since then, a great deal of effort has been devoted to elucidating the characteristics and regulatory mechanisms of ferroptosis. Ferroptosis can be induced through either the extrinsic or intrinsic pathway (66, 67). The extrinsic pathway, also known as the transporter-dependent pathway, is triggered through decreased cystine uptake or increased iron uptake (**Figure 3**). The intrinsic pathway, also called the enzyme-regulated pathway, is primarily induced by the inhibition of intracellular antioxidant enzymes (e.g., glutathione peroxidase 4 (GPX4)).

Lipid peroxidation is a key initiator of ferroptosis (68). Under energetic or oxidative stress, free polyunsaturated fatty acids (PUFAs) are catalyzed by acyl-CoA synthetase long-chain family member 4 (ACSL4), lysophosphatidylcholine acyltransferase 3 (LPCAT3) and arachidonate lipoxygenases (ALOXs) to produce PUFA-containing phospholipids (PUFA-PLs) (69) (**Figure 3A**). Membrane electron transfer proteins are involved in reactive oxygen species (ROS) production during lipid peroxidation. PUFA-PL is then converted into toxic lipid peroxides (PUFA phospholipid hydroperoxides (PUFA-PL-OOH)) with the assistance of lipoxygenases (LOXs) and eventually induces ferroptotic cell death (70).

System xc^-^, a cystine/glutamate transporter, is composed of solute carrier family 7 member 11 (SLC7A11) and solute carrier family 3 member 2 (SLC3A2) (71). System xc^-^ acts to sustain the intracellular content of glutathione (GSH) through regulation of cystine uptake (72). Extracellular cystine is exchanged with glutamate to enter the cell through system xc^-^ and is then converted into cysteine (73) (**Figure 3B**). Cysteine promotes the synthesis of GSH. GPX4 can transform GSH into oxidized glutathione (GSSG) and simultaneously reduce toxic lipid hydroperoxide (LOOHs) to nontoxic lipid alcohols (L-OHs), thus inhibiting the generation of lipid-based ROS and protecting cells from ferroptosis (60). Blockade of system xc^-^ by erastin, GSH deficiency, or a GPX4 inhibitor (e.g., RSL3) induces ferroptotic cell death by promoting lipid-ROS production (74).

Accumulated intracellular iron is another contributor to ferroptosis (75). In iron metabolism, extracellular ferric iron (Fe^3+^) enters the cell through transferrin and transferrin receptor 1 (TFR1) (76) (**Figure 3C**). In cells, Fe^3+^ is released from transferrin and is reduced to ferrous iron (Fe^2+^) by the iron reductase six-transmembrane epithelial antigen of prostate 3 (STEAP3). Fe^2+^ is transferred into a labile iron pool under the action of divalent metal transporter 1 (DMT1) or zinc-iron regulatory protein (ZIP) family 8/14 (77, 78). Labile iron is generally stored in the iron-storage protein ferritin, thereby restricting the high reactivity of iron and inhibiting ROS generation (79). The imbalance in iron metabolism can lead to ferroptosis (80). In practice, nuclear receptor coactivator 4 (NCOA4) binds to ferritin and transports it to the lysosome for degradation, which results in the release of free Fe^2+^ (81). Excess Fe^2+^ contributes to ROS formation and consequent oxidative stress through the Fenton reaction (65). These events drive the peroxidation of lipids, nucleic acids and proteins, culminating in cell ferroptosis (82). In addition to facilitating lipid peroxidation through nonenzymatic mechanisms, excessive intracellular iron can induce ferroptosis by activating ALOX or EGLN prolyl hydroxylase (PHD), which participate in lipid peroxidation and oxygen homeostasis (83, 84).

### 2.4 Overview of necroptosis

**Figure 4:**
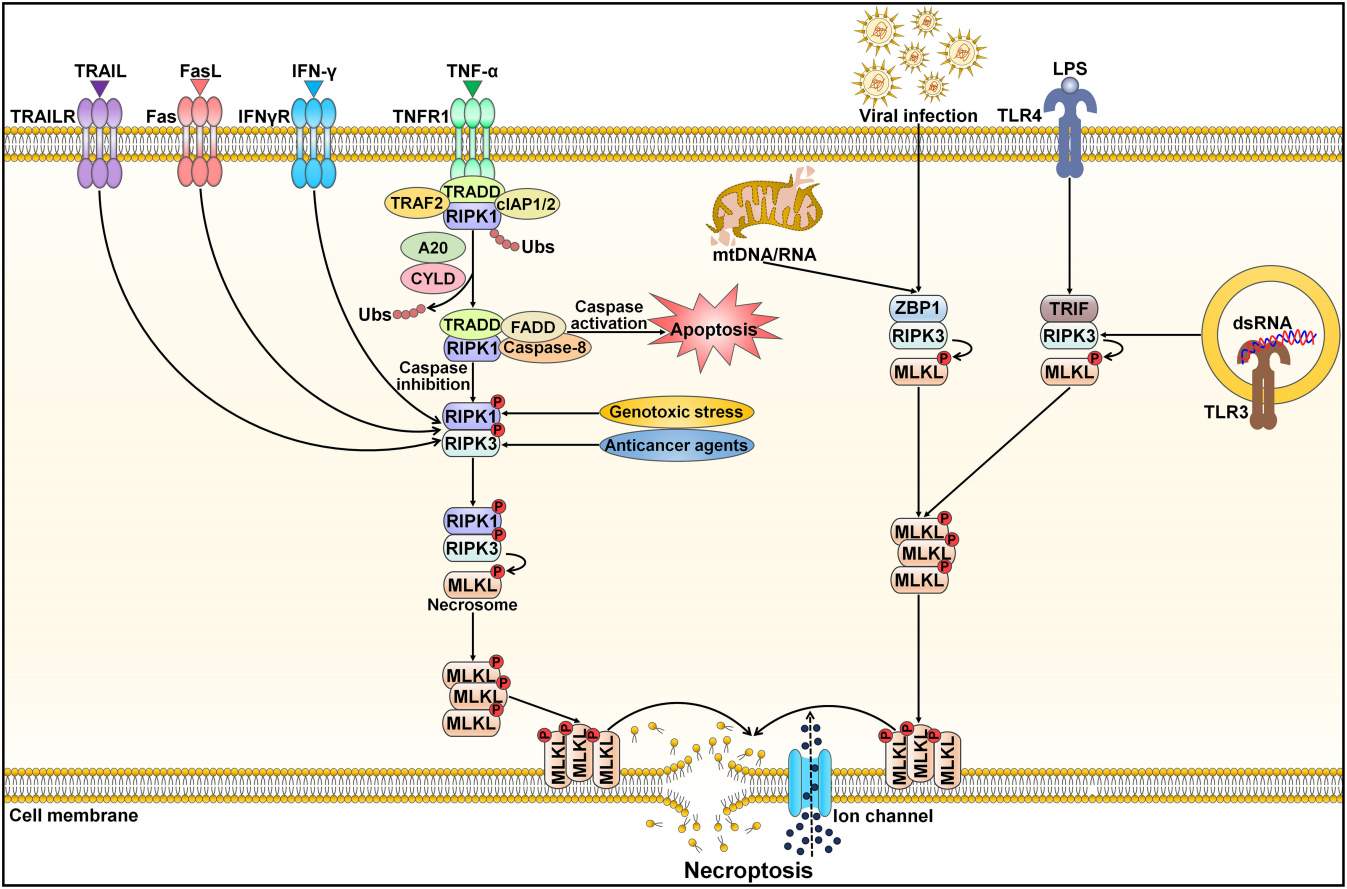
Schematic representation of necroptotic cell death. Necroptosis can be activated by ligand binding to TNF receptor family proteins (e.g., TNFR1, IFNγR, Fas and TRAILR). The most extensively studied subtype of regulated necrosis is TNF-α-induced necroptosis pathway, which relies on the necrosome composed of RIPK1, RIPK3 and MLKL. TNF-α interacts with TNFR1 leading to the structural alteration of TNFR1. A signaling complex (complex I) is assembled at the cytoplasmic domains of TNFR1 and comprises TRAF2, TRADD, RIPK1 and cIAP1/2. Several factors, especially the ubiquitination status of RIPK1 and the activation state of caspase-8, influence whether or not the cell adopts a necroptotic fate. Polyubiquitinated RIPK1 contributes to the stabilization of complex I and triggers an alternative pathway that facilitates cell survival via MAPK and NF-κB signals. When cIAPs are degraded or inhibited, ubiquitination of RIPK1 is blocked, and A20 and CYLD induce RIPK1 deubiquitination. This promotes RIPK1 dissociation from the cell membrane and induces the activation of cell death pathways (apoptosis and necroptosis). RIPK1 deubiquitination facilitates the generation of a cytosolic apoptotic complex (complex II) that includes TRADD, FADD, RIPK1 and caspase-8. Caspase-8 triggers apoptosis and prevents necroptosis by cleaving RIPK1 and RIPK3. In the absence of caspase-8, RIPK1 recruits and phosphorylates RIPK3, following which activated RIPK3 phosphorylates MLKL and results in its oligomerization. The MLKL oligomer migrates to the cell membrane and generates large pores that contribute to necroptotic cell death by allowing ion influx, and cell swelling and lysis. Moreover, necroptosis can be initiated by other inducers, including genotoxic stress, anticancer agents, nucleic acids (DNAs and RNAs) released from damaged mitochondria, viral infection, LPS and dsRNA. After sensing cytosolic mtDNA/mtRNA or virus-derived RNA, ZBP1 induces necroptosis via the RIPK3/MLKL axis. TLR3 and TLR4 are activated by dsRNA and LPS, respectively. Thereafter, TLR stimulation triggers RIPK1-independent necroptosis via TRIF. TRAIL, tumor necrosis factor-related apoptosis-inducing ligand; TRAILR, tumor necrosis factor-related apoptosis-inducing ligand receptor; FasL, Fas ligand; IFN-γ, interferon-γ; IFNγR, interferon-γ receptor; TNF-α, tumor necrosis factor-α; TNFR1, tumor necrosis factor-α receptor 1; TRAF2, tumor necrosis factor receptor-associated factor 2; TRADD, tumor necrosis factor receptor-associated death domain; cIAP1/2, cellular inhibitor of apoptosis protein 1/2; RIPK1, receptor-interacting protein kinase 1; Ubs, ubiquitins; CYLD, cylindromatosis; FADD, Fas-associated protein with death domain; RIPK3, receptor-interacting protein kinase 3; MLKL, mixed lineage kinase domain-like protein; mtDNA/RNA, mitochondrial DNA/RNA; ZBP1, Z-DNA binding protein 1; LPS, lipopolysaccharide; TLR4, Toll-like receptor 4; TRIF, Toll/interleukin-1 receptor domain-containing adapter-inducing interferon-β; dsRNA, double-stranded RNA; TLR3, Toll-like receptor 3.

Necroptosis, also referred to as programmed necrosis, is a necrosis-like form of cell death driven by RIPK1, RIPK3 and mixed lineage kinase domain-like protein (MLKL) (85, 86). Necroptosis has been closely associated with diverse pathologies, including cancer and cardiovascular diseases (87, 88). It shares similar morphological features with necrosis, such as a translucent cytoplasm, swelling of organelles, plasma membrane rupture, mitochondrial dysfunction, increased cell volume, cell lysis, extravasation of intracellular contents and chromatin condensation (89). Unlike apoptosis and pyroptosis, necroptosis is initiated by caspase-independent signaling pathways (90) (**Figure 4**). Necroptosis can be activated by a plethora of internal and external stimuli, including the TNF superfamily members (e.g., Fas ligand (FasL), TNF-α and TNF-related apoptosis-inducing ligand (TRAIL)), interferon-γ (IFN-γ), bacteria, DAMPs, endoplasmic reticulum (ER) stress, hypoxia, LPS, metabolic and genotoxic stresses, ROS, viral infection and chemotherapeutic agents (91, 92). Necroptosis occurs after necroptotic activators are detected by cytosolic nucleic acid sensors, death receptors, IFN receptors, PRRs, T cell receptors (TCRs) and Toll-like receptors (TLRs) (88, 93). The most well-characterized necroptosis mechanism is triggered by ligation of the death receptor TNFR1 to its ligand TNF-α (92). TNFR1 then undergoes conformational changes that allow it to recruit a series of proteins, such as cellular inhibitor of apoptosis protein 1 (cIAP1), cIAP2, linear ubiquitin chain assembly complex (LUBAC), RIPK1, TRAF2, TRAF5 and TRADD (94). This short-lived membrane signaling complex is known as complex I. LUBAC and cIAPs induce RIPK1 ubiquitination, which creates stable complex I and triggers an alternative pathway that facilitates cell survival via mitogen-activated protein kinase (MAPK) and NF-κB signalings (95). When cIAPs are degraded or inhibited, ubiquitination of RIPK1 is blocked, and A20 and cylindromatosis (CYLD) mediate RIPK1 deubiquitination (96, 97). This promotes RIPK1 dissociation from the cell membrane and leads to activation of cell death pathways (e.g., apoptosis and necroptosis) (98). RIPK1 deubiquitination encourages the development of a cytosolic apoptotic complex (complex II) that is composed of caspase-8, FADD, RIPK1 and TRADD (99). Caspase-8 triggers apoptosis and impedes necroptosis by cleaving RIPK1 and RIPK3 (100). In conditions where caspase-8 is inhibited or RIPK3 is overexpressed, complex II does not initiate the apoptosis pathway, but rather, it induces necroptotic cell death (101). In the absence of caspase-8 activity, RIPK1 recruits and phosphorylates RIPK3, leading to assembly of the necrosome (102). Following this, the RIPK1/RIPK3 complex facilitates phosphorylation and oligomerization of MLKL (103). With the ability to interact with lipids, oligomerized MLKL translocates toward the cell membrane, where it creates large pores and causes uncontrollable release of cellular contents (e.g., DAMPs and inflammatory cytokines) (104). The MLKL oligomer combines with ion channels to disrupt cellular ion homeostasis, which then drives osmotic cell membrane rupture and ultimately induces necroptosis (89). In addition to the TNF-mediated necroptosis mechanism, other pathways (nonclassical necroptotic pathways) that elicit necroptosis have been reported (98). For instance, Z-DNA-binding protein 1 (ZBP1) and Toll/IL-1 receptor (TIR) domain-containing adapter-inducing IFN-β (TRIF) directly bind to RIPK3, leading to RIPK1-independent activation of necroptosis (105, 106).

### 2.5 Overview of autophagy-dependent cell death

**Figure 5:**
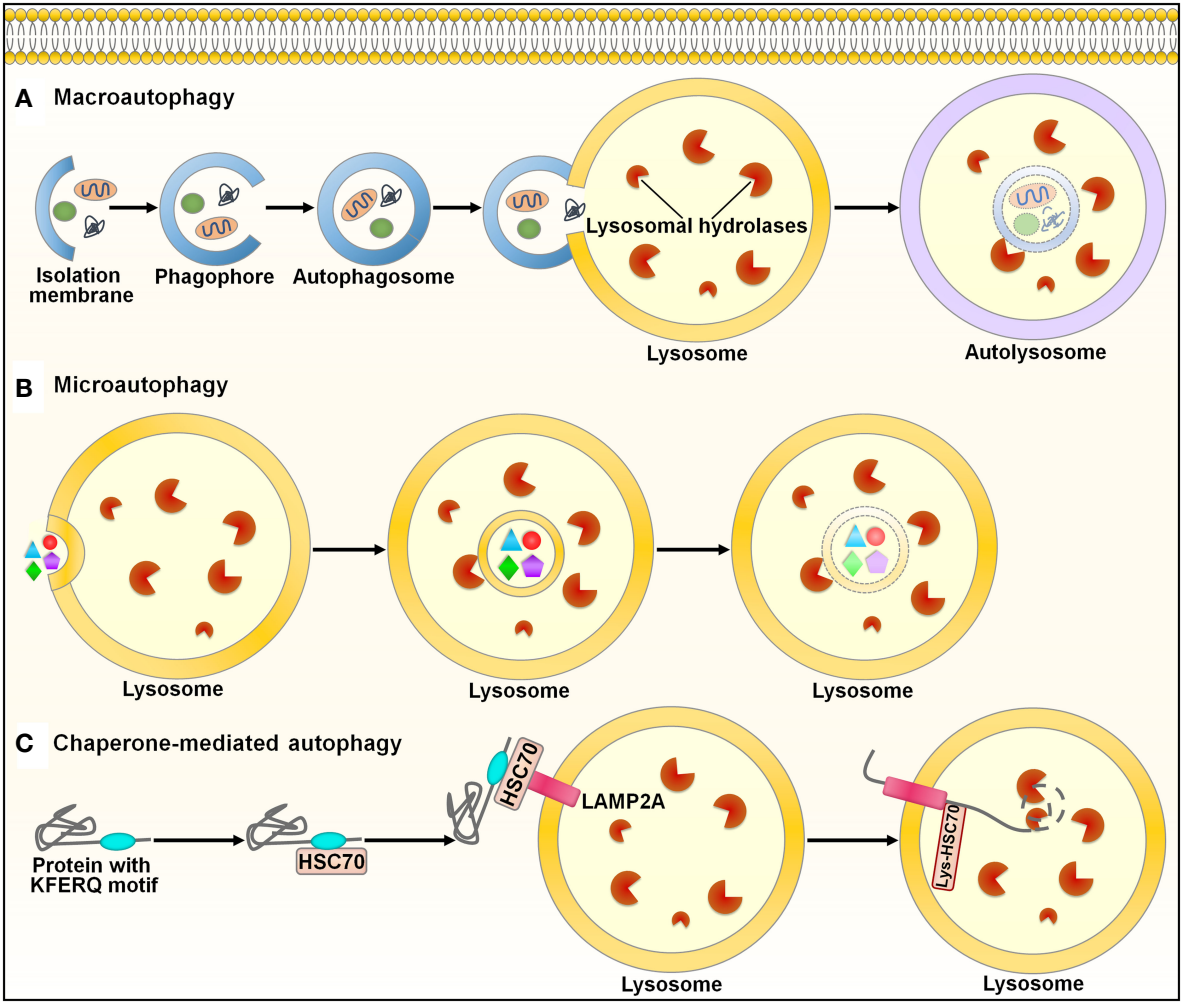
Schematic illustration of the autophagy pathways. Autophagy is categorized into three main types: macroautophagy, microautophay and chaperone-mediated autophagy. **(A)** Macroautophagy. This pathway is initiated with the nucleation of a double-membraned structure, named phagophore. The phagophore is elongated to enclose cytoplasmic contents, leading to the formation of a double-membrane vesicle called autophagosome. The autophagosome subsequently fuses with the lysosome to form an autolysosome, where degradation of cargo contents occurs. **(B)** Microautophagy. Microautophagy involves direct incorporation of cytoplasmic components into the lysosome for degradation. **(C)** Chaperone-mediated autophagy. In this pathway, the cytosolic chaperone HSC70 recognizes proteins containing the KFERQ-like motif. The HSC70-target protein complex is translocated to the lysosome through binding to the LAMP2A receptor on the lysosomal membrane. CMA-targeted proteins undergo degradation within the lysosome. HSC70, heat shock cognate protein 70; LAMP2A, lysosome-associated membrane protein 2A.

Autophagy (self-eating) is an evolutionarily conserved mechanism for the degradation and recycling of cytoplasmic materials (e.g., organelles, lipids and proteins) via lysosomal digestion (107). Autophagy is engaged in many biological processes, such as development and differentiation (108). There are three main types of autophagy in mammals: macroautophagy, microautophagy and chaperone-mediated autophagy (CMA) (109) (**Figure 5**). Macroautophagy, the most well-known form of autophagy, is a nonselective cellular process. This autophagic process starts with the nucleation of a double-membraned structure termed phagophore (110) (**Figure 5A**). The phagophore is elongated to enclose cytoplasmic contents, which leads to the formation of a double-membrane vesicle called autophagosome. The autophagosome subsequently fuses with the lysosome to form an autolysosome, where degradation of cargo contents occurs. Microautophagy involves the direct incorporation of cytoplasmic components into the lysosome or late endosome for degradation (111) (**Figure 5B**). Endosomal sorting complexes required for transport (ESCRT) machinery is needed for the membrane fission step (112). In the CMA pathway, the cytosolic chaperone heat shock cognate protein 70 (HSC70) recognizes CMA-targeted proteins harboring a pentapeptide motif (113) (**Figure 5C**). The HSC70-target protein complex is translocated to the lysosome through binding to the lysosome-associated membrane protein 2A (LAMP2A) receptor on the lysosomal membrane. CMA-targeted proteins undergo degradation within the lysosome. In all types of autophagy, the resultant products of the degradation process are recycled into the cytosol and can be reutilized in a variety of biological processes, such as adenosine triphosphate (ATP) generation, and nucleotide, protein and lipid biosynthesis (114).

The role of autophagy in initiating cell death is highly contextual. It is still unclear how autophagy-dependent processes cause cell death. Autosis, a specific form of autophagy-dependent cell death, is critically dependent on plasma membrane Na^+^/K^+^-ATPase (115). Moreover, cell death is promoted when feedback mechanisms to suppress autophagy are interrupted (116). Cells undergoing autophagy-dependent cell death are characterized by the generation of abundant autophagic vacuoles/vesicles. Thus, hyperactivation of autophagy may induce cell death. Likewise, degraded cytoplasmic components, intracellular membranes and organelles are usually detected in cells undergoing autophagy, which suggests that deprivation of cytoplasmic materials upon persistent autophagy contributes to cell death. It is still unknown whether the degradation of specific organelles is the primary cause of autophagy-dependent cell death or whether excessive degradation of cellular components by autophagy triggers cell death. Since autophagy mainly relies on lysosome function, lysosome-mediated bulk degradation of cytoplasmic contents can induce cellular demise (117). The involvement of the lysosome in autophagy-dependent cell death therefore requires further investigation. Ubiquitination is important for the engulfment of intracellular cargos in selective autophagy (118). Cargo receptors combine with specific ubiquitinated substrates to guide the selective packaging of cargos into autophagosomes (119). The ubiquitin system may play a role in autophagy-dependent cell death, but further investigation is required to substantiate and expand this assumption. The autophagic machinery may selectively degrade prosurvival factors to elicit cell death. Reportedly, autophagy promoted the degradation of the anti-apoptotic dBruce to drive caspase-dependent apoptosis in nurse cells during late *Drosophila melanogaster* oogenesis (120). The molecular machinery necessary for autophagy-dependent cell death may be different from that favoring cell survival. The rate of autophagic flux, the duration of activated autophagy and the fate of engulfed components may bring about different requirements for autophagy machinery components. Altogether, autophagy-dependent cell death seems to be coordinated differently from prosurvival autophagy. In addition, various signaling pathways, such as the phosphoinositide-3-kinase (PI3K) pathway and MAPK signaling pathway, may regulate autophagy-dependent cell death (121, 122). The detailed mechanisms regulating autophagy-dependent cell death have not yet been fully defined. Future research efforts are necessary to better understand the modulatory mechanisms of autophagy-dependent cell death.

## 3 Crosstalk between programmed cell death and tumor microenvironment

Increasing evidence has shown that various types of PCD including apoptosis, autophagy-dependent cell death, pyroptosis and ferroptosis can enhance antitumor immunity via stimulation of immune-promoting cells or release of proinflammatory mediators. In some cases, PCD also limits antitumor immune responses through elimination of antitumor immune cells or activation of immunosuppressive cells.

### 3.1 Apoptosis in tumor immunity

Several studies have demonstrated that apoptosis can stimulate antitumor immune cells (**Table 1**). High expression of STAT3 was associated with chemotherapy resistance and immune evasion of hepatocellular carcinoma (HCC) cells (123). STAT3 absence aggravated therapeutically induced-ER stress-mediated apoptosis in HCC. The DNA derived from apoptotic HCC cells stimulated the cyclic guanosine monophosphate-adenosine monophosphate synthase (cGAS)/stimulator of interferon genes (STING) signaling cascade in CD103^+^ DCs and triggered type I IFN (IFN-I) production, which eventually augmented the antitumor function of CD8^+^ T and NK cells. Accordingly, STAT3 knockdown sensitized HCC cells to the cytotoxic agent sorafenib. Insulin-like growth factor-2 mRNA-binding protein 1 (IGF2BP1) recognized m^6^A target transcripts, including *c-MYC* and *PTEN* (155). IGF2BP1 acted as an oncogenic driver in HCC (124). IGF2BP1 dysfunction efficiently prevented its attachment to m^6^A mRNA targets and led to enhanced apoptosis of HCC cells. These events promoted the activation and intratumoral infiltration of various immune cells (e.g., macrophages, NK cells, CD4^+^ T cells and CD8^+^ T cells) and restricted PD-L1 expression to block immunosuppression.

**Table 1:** Overview of the effects of programmed cell death on tumor immunity.

Programmed cell death	Cancer type	Action	Effect on antitumor immunity	Reference
Apoptosis	Hepatocellular carcinoma	Activate the cGAS/STING signaling pathway in CD103^+^ DCs; increase IFN-I production	Promotion	(123)
	Hepatocellular carcinoma	Activate macrophages, NK cells, CD4^+^ T cells and CD8^+^ T cells	Promotion	(124)
	Mammary tumor	Induce accumulation of immunosuppressive cytokines and leukocytes	Inhibition	(125)
	Hepatocellular carcinoma	Activate tumor-supportive immune cells; inhibit antitumor immune cells	Inhibition	(126)
	Breast cancer	Facilitate NET formation	Inhibition	(127)
	Basal cell carcinoma	Induce NK cell dysfunction	Inhibition	(128)
Autophagy-dependent cell death	Hepatocellular carcinoma	Repress macrophage polarization into M2 phenotype	Promotion	(129)
	Lung adenocarcinoma	Inhibit CD8^+^ CTL function	Inhibition	(130)
	Prostate cancer	Restrict functional T cell activation	Inhibition	(131)
Pyroptosis	Melanoma	Activate DCs; promote T cell expansion	Promotion	(132)
	Colon cancer	Facilitate DC maturation and T cell activation	Promotion	(133)
	Melanoma, colon cancer, mesothelioma, breast cancer, lung cancer	Enhance CTL infiltration	Promotion	(134)
	Breast cancer, melanoma, lung cancer	Enhance CTL infiltration	Promotion	(135)
	Pancreatic cancer	Favor macrophage polarization into M1 phenotype; induce DC maturation and CD8^+^ CTL activation	Promotion	(136)
	Prostate cancer	Enhance intratumoral infiltration of CD8^+^ T cells	Promotion	(137)
Ferroptosis	Glioma	Foster macrophage polarization into M2 phenotype; inhibit CD8^+^ T cell activation	Inhibition	(138)
	Pancreatic ductal adenocarcinoma	Promote macrophage polarization into M2 phenotype	Inhibition	(139)
	Lymphoma, colon cancer, lung carcinoma	Increase secretion of immunosuppressive molecules	Inhibition	(140)
	Multiple myeloma	Repress cytotoxic cytokine production; restrict T cell function	Inhibition	(141)
	Lung squamous cell carcinoma	Enhance CD8^+^ T cell function	Promotion	(142)
	Gastrointestinal cancer	Reduce CAF infiltration	Promotion	(143)
	Head and neck squamous cell carcinoma	Decrease MDSCs and M2 macrophages; increase CD4^+^ and CD8^+^ T cells	Promotion	(144)
	Hepatocellular carcinoma	Favor M1 macrophage production; induce CD8^+^ T cell activation	Promotion	(145)
Necroptosis	Melanoma, lung carcinoma, lymphoma	Foster antigen uptake and APC activation	Promotion	(146)
	Triple-negative breast cancer	–	Promotion	(147)
Immunological cell death	Hepatocellular carcinoma	Promote DC maturation and CD8^+^ T cell infiltration	Promotion	(148)
	Non-small cell lung cancer	Elicit cytokine response; increase CD8^+^ T cell infiltration	Promotion	(149)
	Neuroblastoma	Promote antigen cross-presentation; activate tumor-specific CD8^+^ T cells	Promotion	(150)
	Glioblastoma multiforme	Facilitate DC activation	Promotion	(151)
	Non-small cell lung cancer, melanoma	Promote antigen cross-presentation; induce CD8^+^ T cell activation	Promotion	(152)
	Melanoma, prostate cancer, glioma	Enhance CD8^+^ T cell function	Promotion	(153)
	Breast cancer, melanoma, osteosarcoma, prostate cancer, rectal cancer	Increase cancer cell adjuvanticity and immunogenicity; facilitate CTL activation	Promotion	(154)

Apoptosis contributes to the reshaping of an immunosuppressive TME. Apoptotic cells are commonly cleared from tissues via efferocytosis (156). The clearance of apoptotic cells by efferocytosis prevents these cells from undergoing secondary necrosis and secreting proinflammatory factors. Efferocytosis expeditiously eradicated lapatinib-induced apoptotic cancer cells from mammary tumors *in vivo* (125). This process upregulated immunosuppressive cytokines (e.g., IL-10, IL-13 and TGF-β1) and recruited immunosuppressive leukocytes (e.g., myeloid-derived suppressor cells (MDSCs) and regulatory T cells (Tregs)) to the tumors. Thus, cancer cell efferocytosis following chemotherapy imparted tolerance to cancer cells and enabled them to escape treatment-induced apoptosis. Inhibition of efferocytosis triggered secondary necrosis of apoptotic cells but failed to prevent the aforementioned immunosuppressive alternations in response to cancer cell death. Mechanistically, necrosis secondary to impaired efferocytosis stimulated IFN-γ-inducible expression of indoleamine 2,3-dioxygenase 1 (IDO1), culminating in immunosuppression and cancer development. Combined suppression of efferocytosis and IDO1 dampened cancer cell death-induced immunosuppression and restricted mammary tumor progression in murine models. Altogether, apoptotic and necrotic cell death pathways affect cancer progression through different mechanisms. Additional work is warranted to gain a fundamental understanding of the intricate communication between diverse cell death pathways and the host immune system. A recent study showed that apoptosis, ferroptosis and pyroptosis were common types of cell death processes in the TME of HCC (126). These cell death pathways were cooperatively involved in establishing an immunosuppressive TME, given their close association with increased intratumoral infiltration of tumor-supportive immune cells (e.g., activated mast cells and Tregs) as well as decreased infiltration of antitumor immune cells (e.g., γδ T cells, monocytes and neutrophils). The Treg-like Vδ1^+^ γδ T cell population was dominant over the cytotoxic Vδ2^+^ population and comprised the major γδ T cell subset in HCC. Adoptive transfer of allogeneic Vδ2^+^ γδ T cells may represent an effective immunotherapeutic approach for HCC.

Apoptosis can facilitate cancer immune evasion by repressing antitumor immunity. Intrinsic apoptosis in cancer cells affects the ability of the TME to escape antitumor immunity. Tumor-associated neutrophil-released neutrophil extracellular traps (NETs) repressed the function of effector immune cells (157). Reportedly, apoptotic breast cancer cells secreted spermidine to foster NET formation in a pannexin 1 (Panx1) channel-dependent manner, which attenuated antitumor immunity (127). Suppression of spermidine synthesis inhibited breast cancer growth *in vivo*. Thus, cancer cells remodel the tumor immune microenvironment to evade immunosurveillance through the release of metabolites from intrinsic apoptotic cells. The effects of other metabolites derived from apoptotic cells on the tumor immune microenvironment deserve further study. Basal cell carcinoma (BCC)-expressed CD200 was released into the TME as soluble CD200 (sCD200) (128). In the TME, sCD200 bound the respective receptor on NK cells to block the MAPK signaling pathway, which promoted peroxisome proliferator-activated receptor γ (PPARγ)-mediated gene transcription of Fas death receptor family members (FasL, Fas and FADD). This in turn contributed to autoregulatory activation-induced NK cell apoptosis. Blocking sCD200-mediated suppression of the MAPK or PPARγ signaling cascade promoted the survival and tumor-killing activity of NK cells. Therefore, BCC cells can remodel the TME via sCD200 release, which leads to NK cell exhaustion and cancer immune evasion. It is intriguing whether sCD200-mediated NK cell dysfunction also constitutes an immune escape mechanism in other cancer types.

### 3.2 Autophagy-dependent cell death in tumor immunity

Autophagy-dependent cell death plays contradictory roles in the regulation of the tumor immune microenvironment. Autophagy-dependent cell death is involved in macrophage polarization. For example, HCC cells polarized cocultured macrophages into the M2-like phenotype (129). Blockade of autophagy-dependent cell death in macrophages inhibited the NF-κB pathway by promoting ubiquitination-mediated degradation of TGF-β-activated kinase-binding protein 3 (TAB3), which facilitated M2-like macrophage polarization. These effects could be impeded by activation of autophagy-dependent cell death. Interference with M2 type macrophage polarization may be a promising treatment strategy alone or in combination with cancer immunotherapy.

Autophagy-dependent cell death can affect the response to immunotherapy through the coordination of adaptive immune cells. Loss of AT-rich interaction domain containing protein 1A (ARID1A) suppressed autophagy-dependent cell death by inducing the epidermal growth factor receptor (EGFR)/PI3K/protein kinase B (Akt)/mammalian target of rapamycin (mTOR) pathway (130). This caused increased production of IFN-I and enhanced infiltration of CD8^+^ cytotoxic T lymphocytes (CTLs), which resulted in an improved response to anti-programmed cell death protein-1 (PD-1)/anti-PD-L1 in patients with *EGFR*-mutant lung adenocarcinoma (LUAD). The multityrosine kinase inhibitor (MTKI) ESK981 exhibited an autophagy-inhibitory property, as it downregulated the lipid kinase PIKfyve (131). ESK981-mediated inhibition of autophagy-dependent cell death promoted C-X-C motif chemokine (CXCL10) secretion via the IFN-γ pathway and enhanced functional T cell infiltration, which conferred increased sensitivity to anti-PD-1 immunotherapy in preclinical models of prostate cancer. Collectively, inhibition of autophagy-dependent cell death can turn tumors from an immunologically “cold” state to an inflamed “hot” state, contributing to priming of the tumor immune microenvironment.

### 3.3 Pyroptosis in tumor immunity

Recently, the role of the pyroptotic pathway in antitumor immunity has been revealed (**Table 1**). The combination of the B-raf proto-oncogene, serine/threonine kinase (BRAF) inhibitors and mitogen-activated extracellular signal-regulated kinase (MEK) inhibitors (BRAFi + MEKi) impeded the extracellular signal-regulated kinase 1/2 (ERK1/2) signaling cascade in melanoma cells, which induced the activation of caspase-3 and resulted in GSDME-executed pyroptosis as well as extravasation of DAMPs (132). Pyroptotic cancer cell-derived DAMPs activated DCs to promote T cell expansion, which led to tumor regression. Conversely, GSDME-knockdown melanoma displayed defective DAMP release and reduced intratumoral T cell infiltration during BRAFi + MEKi treatment. ERK1/2 inhibition produces intense antitumor immune responses through the induction of GSDME-mediated pyroptosis. BRAFi + MEKi-resistant melanoma cells did not undergo pyroptosis but were susceptible to pyroptosis-inducing chemotherapy. Pharmacological reinduction of pyroptosis retarded the growth of BRAFi + MEKi-resistant melanoma cells and might represent an effective salvage option for targeted therapy-resistant melanoma. *Listeria monocytogenes* (*Lmo*)-based immunotherapy (*Lmo*@RBC) induced GSDMC-dependent pyroptosis in colon cancer cells (133). Proinflammatory factors released by pyroptotic cancer cells promoted DC maturation and T cell activation, which culminated in efficient inhibition of primary and metastatic tumors. These findings provide evidence for the potential use of this live bacterial vaccine in cancer immunotherapy. Oncolytic parapoxvirus ovis (ORFV) promoted intratumoral infiltration of CTLs and exhibited tumor killing effects *in vivo* by inducing GSDME-dependent pyroptosis in cancer cells (134). Moreover, ORFV also sensitized immune-cold tumors to immune checkpoint blockade (ICB) therapy. Blocking GSDME-mediated pyroptosis abolished these effects. Similarly, oncolytic vesicular stomatitis virus (VSV) induced cancer cell pyroptosis by activating GSDME (135). VSV treatment suppressed tumor growth by attracting CTLs to the tumor site. Knockdown of GSDME dampened VSV-mediated tumor-antagonizing effects and reduced the ability of VSV to stimulate antitumor immune responses. Furthermore, VSV therapy increased the sensitivity of immunologically “cold” tumors to anti-PD-1 treatment. The combination of oncolytic virus (OV)-based approaches and immunotherapy might be a powerful treatment paradigm against cancer. Membrane anchoring photosensitizer-driven pyroptosis of pancreatic cancer cells reshaped the TME by fostering M1 macrophage polarization, DC maturation and CD8^+^ CTL activation (136). Pyroptosis-mediated reprogramming of the tumor immune microenvironment turned poorly immunogenic tumors into T cell-inflamed and -tumoricidal microenvironments, which led to the inhibition of primary and metastatic tumors. These results highlighted the critical role of pyroptosis in light-controlled antitumor immunity.

The E3 ubiquitin ligase cell division cycle 20 homolog (CDC20) has been recognized as an oncogenic driver (158). A recent study revealed that CDC20 exerted an inhibitory effect on antitumor immune responses and facilitated prostate cancer pathogenesis (137). Mechanistically, CDC20 enhanced ubiquitination-mediated GSDME degradation to prevent pyroptosis in prostate cancer cells, hence reducing intratumoral infiltration of CD8^+^ T cells. Consequently, CDC20 inhibition improved the response to anti-PD-1 in murine models of prostate cancer. Activated immune cells can in turn coordinate cancer cell pyroptosis. It was reported that blockade of cytotoxic T lymphocyte-associated protein-4 (CTLA-4) stimulated CD8^+^ T cells and upregulated the expression of IFN-γ and TNF-α in the TME, which induced the STAT1/interferon regulatory factor 1 (IRF1) pathway to trigger pyroptosis in head and neck squamous cell carcinoma (HNSCC) (159). The impact of the tumor immune microenvironment on cancer cell pyroptosis warrants further study.

### 3.4 Ferroptosis in tumor immunity

Ferroptosis serves as a key mechanism that regulates the crosstalk between cancer cells and the TME. Reportedly, ferroptosis was the most enriched PCD pathway in glioma (138). Increased ferroptosis was linked to exacerbated immunosuppression and poor patient outcomes. Ferroptosis fostered the recruitment and polarization of tumor-associated macrophages (TAMs) into an immunosuppressive M2-like phenotype and reduced the cytotoxic activity of CD8^+^ T cells. Pharmacological inhibition of ferroptosis in combination with ICB produced a synergistic therapeutic outcome in glioma-bearing mice. Autophagy-dependent ferroptosis induced by oxidative stress promoted the transfer of KRAS^G12D^ from pancreatic ductal adenocarcinoma (PDAC) to macrophages through exosomes (139). KRAS^G12D^ then drove macrophage polarization into the M2-like pro-tumor phenotype by inducing fatty acid oxidation. Inhibition of KRAS^G12D^ release and uptake prevented macrophage-mediated PDAC progression *in vivo*. Polymorphonuclear (PMN)-MDSCs in the TME underwent spontaneous ferroptosis, which increased the liberation of immunosuppressive molecules (e.g., peroxidized lipids) from ferroptotic cells, leading to compromised CD8^+^ T cell-mediated immune responses and cancer progression (140). The secretion of immunosuppressive factors preceded PMN-MDSC cell death during ferroptosis. Blockade of ferroptosis prevented PMN-MDSC death and the release of immunosuppressive molecules, which shifted PMN-MDSCs into classical nonsuppressive PMNs. Furthermore, pharmacological inhibition of ferroptosis magnified the anticancer activity of anti-PD-1 in experimental animal models. CD36, a scavenger receptor involved in lipid metabolism, promoted ferroptosis in CD8^+^ T cells by facilitating the uptake of fatty acids to induce lipid peroxidation (141). CD36-mediated ferroptosis inhibited cytotoxic cytokine production and dampened T cell function in multiple myeloma (141). Blocking ferroptosis in CD8^+^ T cells effectively restored their tumor-killing effects and augmented the anticancer efficacy of anti-PD-1 therapy.

In contrast, ferroptosis functions to induce antitumor immune responses. For instance, the activation of ferroptosis mediated the tumor-suppressive role of resveratrol in lung squamous cell carcinoma (LUSC) by enhancing the cytotoxic effect of CD8^+^ T cells (142). Anoctamin 1 (ANO1) promoted TGF-β secretion by gastrointestinal (GI) cancer cells, which attracted cancer-associated fibroblasts (CAFs) into the TME by blocking ferroptosis in a PI3K/Akt signaling-dependent manner (143). CAFs impaired CD8^+^ T cell-mediated immunity via the CAF-related secretome, thus enhancing resistance to anti-PD-1 immunotherapy and accelerating GI cancer progression. ANO1-mediated ferroptosis inhibition represents a vital mechanism underlying TME reprogramming and immunotherapy resistance in GI cancer. Moreover, ferroptosis can reverse the immunosuppressive microenvironment. RSL3-induced ferroptosis in HNSCC decreased the number of MDSCs and M2 macrophages and increased the number of tumor-infiltrating CD4^+^ and CD8^+^ T cells in the TME (144). Ferroptosis-inducing agents may be an attractive therapeutic option for HNSCC. Apolipoprotein C1 (APOC1) was found to be involved in cancer pathogenesis (160). Overexpression of APOC1 in TAMs in the HCC microenvironment repressed ROS production to inhibit ferroptosis through the regulation of iron and lipid metabolism, which facilitated the transformation of TAMs into the tumor-supportive M2 phenotype and the establishment of a proinflammatory TME (145). Loss of APOC1 promoted the conversion of M2 macrophages into the M1 type and induced the activation of CD8^+^ T cells. *In vivo* experiments demonstrated that APOC1 depletion resulted in immune activation and improved HCC sensitivity to anti-PD-1 treatment. Ferroptosis may act in an opposite manner in cancer progression through a myriad of mechanisms, such as promotion of cancer cell death, mobilization of cytotoxic T cells and induction of an immunosuppressive phenotype. Induction of ferroptosis significantly inhibits cancer development. However, in some cancers, the immunosuppressive effect of ferroptosis occupies a dominant position, which may be an important explanation for the limited therapeutic potential of ferroptosis inducers in preclinical models. The effect of ferroptosis on carcinogenesis and tumor immunity may differ depending on the type of cells in which this process occurs. The therapeutic benefit of ferroptosis-regulating agents must therefore be carefully evaluated. Continual studies will be required to maximize the efficacy of ferroptosis modulation by curbing the inhibition of cancer cell death and surmounting the immunosuppressive phenotype. Cell-specific delivery of ferroptosis-inducing or -suppressing agents may be a clinically relevant therapeutic approach. Undoubtedly, elucidating the detailed mechanisms of ferroptosis in cancer-TME interaction networks will offer valuable opportunities to develop innovative therapeutic approaches for cancer intervention.

In addition, immune cells can regulate ferroptosis in cancer cells. ICB (e.g., anti-CTLA-4 and anti-PD-L1)-activated CD8^+^ T cells reduced the expression of two subunits (SLC3A2 and SLC7A11) of the glutamate-cystine antiporter system xc^-^ and limited cystine uptake by cancer cells via IFN-γ release (161). As a result, CD8^+^ T cells facilitated lipid peroxidation and ferroptosis in cancer cells. Increased ferroptosis further enhanced the anticancer efficacy of immunotherapy. Moreover, cysteine or cysteine deprivation synergized with ICB to actuate T cell-mediated antitumor immunity and induce cancer cell ferroptosis. The expression of system xc^-^ was shown to be negatively correlated with the CD8^+^ T cell signature, IFN-γ level and clinical outcomes in cancer patients. Accordingly, T cell-induced cancer cell ferroptosis constitutes an anticancer mechanism.

### 3.5 Other cell death pathways in tumor immunity

Ectopic activation of RIPK1/RIPK3 induced necroptosis of fibroblasts within the TME, which contributed to enhanced production of NF-κB-dependent cytokines (146). These events promoted antigen uptake and activation of antigen-presenting cells (APCs) to strengthen tumor-specific CD8^+^ T cell immune responses. RIPK1/RIPK3 activators synergized with ICB to foster systemic tumor control in preclinical animal models. Oncolytic alphavirus M1 induced necroptosis in triple-negative breast cancer (TNBC) (147). This necroptotic virotherapy acted synergistically with doxorubicin (DOX) to inhibit TNBC growth *in vivo*.

ICD, a type of PCD induced by many stressors, involves diverse mechanisms of cell death including apoptosis, ferroptosis, necroptosis and pyroptosis (162). ICD encompasses the emission of DAMPs from dying cancer cells that stimulate antigen cross-presentation and initiate antitumor adaptive immunity (2). Thus, harnessing ICD may represent a promising treatment modality for cancer therapy (163). ICD is emerging as a critical component of chemotherapy-induced antitumor immunity. For instance, oxaliplatin was found to induce immunogenic apoptosis in HCC cells, as evidenced by increased secretion of ICD biomarkers (ATP and high-mobility group box 1 (HMGB1)) from dying cells (148). As expected, oxaliplatin elicited DC maturation and CD8^+^ T cell infiltration. Likewise, sequential treatment with pemetrexed and cisplatin induced ICD in non-small cell lung cancer (NSCLC) cells by activating the STING signaling pathway (149). This treatment contributed to robust cytokine responses and enhanced infiltration of CD8^+^ T cells. Similarly, STING-activating nanoparticles (STING-NPs) effectively activated the STING signaling pathway and induced ICD in neuroblastoma (150). STING-NP-mediated ICD promoted cancer cell phagocytosis and DC cross-presentation of cancer-derived antigens, which eventually triggered tumor-specific CD8^+^ T cell immune responses. Furthermore, STING-NPs retarded cancer growth and induced immunological memory that protected against tumor rechallenge. STING-NPs acted synergistically with PD-L1 blockade in a murine model of neuroblastoma. STING-NPs may have utility as an adjuvant therapy for enhancing responses to immunotherapy in neuroblastoma. Polyglycerol-functionalized nanodiamonds bearing DOX (Nano-DOX) induced autophagy-dependent cell death in glioblastoma multiforme (GBM) and promoted the secretion of cancer antigens and DAMPs by GBM cells, which contributed to DC activation (151). Autophagy-dependent cell death serves as a major mechanism through which Nano-DOX subverts the GBM-associated immunosuppressive microenvironment. These pharmaceuticals combined with ICB may effectively stimulate the adaptive immune system to produce intense tumor-killing effects. OVs are emerging as innovative immunotherapeutic agents and can activate adaptive antitumor immunity through the induction of ICD, such as immunogenic apoptosis, autophagy-dependent cell death, pyroptosis and necroptosis (152). OVs have been engineered to facilitate ICD through mechanisms involving insertion or deletion of death pathway-regulating genes. Genetically modified OVs act to skew infected cancer cells toward specific death pathways for increased immunogenicity. OV-based immunotherapy in combination with standard therapeutic regimens may be effective in potentiating antitumor immunity and improving therapeutic benefits. For instance, the oncolytic virus M1 was shown to be capable of eliciting ICD in cancer (153). M1 promoted DC cell activation to actuate antitumor CD8^+^ T cell responses and contributed to long-term antitumor immune memory *in vivo*. M1 treatment also increased the efficacy of anti-PD-L1 therapy. In addition, many types of radiation (e.g., nonionizing (ultraviolet) and ionizing radiation) can initiate ICD, thus enhancing the adjuvanticity and immunogenicity of dying cancer cells and activating CTL function (154). Radiation therapy cooperated with conventional immunotherapies (e.g., ICB) to induce systemic antitumor immunity and cancer regression.

## 4 Potential clinical application of targeted therapies against programmed cell death in cancer

Considering the association between PCD and tumor immunity, targeted therapies against PCD have the potential to reinforce the clinical benefit of conventional immunotherapy. Targeting PCD has recently gained substantial attention in the field of cancer immunotherapy. Several clinically used drugs can coordinate tumor immunity through the regulation of PCD pathways in cancer. Paclitaxel, a representative chemotherapeutic agent for lung cancer, induced pyroptosis in lung cancer cells by activating caspase-3 and GSDME (164). Paclitaxel increased the immunogenicity of cancer cells, facilitated antigen presentation, and augmented antitumor immunity (165). As a result, paclitaxel enhanced the efficacy of immunotherapy. BRAFi + MEKi, which are FDA-approved for the treatment of *BRAF* ^V600E/K^-mutant melanoma, activated DCs and tumor-specific T cells in melanoma by triggering GSDME-executed pyroptosis and promoting DAMP release (132). BRAFi + MEKi may improve patient response to immunotherapy, which merits further verification. Sorafenib, a clinically approved anticancer agent, repressed system xc^-^ function and elicited ER stress, which contributed to ferroptotic cell death (166). Sorafenib combined with PD-L1 inhibition induced antitumor T cell immunity and effectively suppressed HCC growth (167). DOX triggered immunogenic cell death (autophagy-dependent cell death and pyroptosis), activated innate/adaptive immune cells, and induced systemic antitumor immunity (168, 169). Cisplatin acted as an inducer of apoptosis and ferroptosis in cancer cells and promoted intratumoral infiltration of CTLs (170, 171). Taken together, the evidence shows that combination treatment with antineoplastic agents and immunotherapy may be superior to each component alone due to adjunctive or synergistic effects.

Furthermore, some clinical trials have shown the therapeutic efficacy of targeted treatments against PCD combined with immunotherapy (172) (**Table 2**). A phase 1 trial showed that NK cell therapy combined with trastuzumab had good tolerance in patients with treatment-refractory HER2-positive solid tumors (173). This combined therapy promoted cancer cell apoptosis and increased NK cell expansion and lymphocytic infiltrates. Phase 2 trials are recommended to further assess the safety and efficacy of NK cell therapy in combination with trastuzumab. Cytokine-induced killer (CIK) cell immunotherapy combined with sintilimab plus chemotherapeutic agents (carboplatin and pemetrexed or paclitaxel) was well tolerated and exhibited antitumor activity in NSCLC patients (174). Tiragolumab plus atezolizumab (31.3%) improved the objective response rate compared with placebo plus atezolizumab (16.2%) in NSCLC patients (175). The tiragolumab plus atezolizumab group (5.4 months) exhibited better median progression-free survival than the placebo plus atezolizumab group (3.6 months). Tiragolumab plus atezolizumab had a manageable safety profile and represented a prospective combination immunotherapy for NSCLC intervention. In a phase 3 trial involving 800 NSCLC patients, durvalumab plus chemotherapy prolonged progression-free survival and overall survival (176). This combined treatment had a tolerable safety profile. NSCLC patients who received pembrolizumab in combination with chemotherapy (gemcitabine, docetaxel or pemetrexed) exhibited higher progression-free survival than patients treated with chemotherapy alone (177). In another study, sitravatinib plus tislelizumab controlled disease progression in the majority (88.5%) of NSCLC patients (178). Nanoparticle albumin-bound paclitaxel (nab-paclitaxel) improved the objective response rate (55.2%) in NSCLC patients following PD-(L)1 inhibitor treatment (179). Lenalidomide is an immune modulator that has been approved for the treatment of relapsed/refractory follicular lymphoma (183). The DNA methyltransferase inhibitor azacytidine activated apoptosis- and immune-related pathways (180). In a phase 1b study, azacytidine in combination with lenalidomide and dexamethasone contributed to an overall response rate of 22% and a clinical benefit response rate of 32% in patients with relapsed or refractory multiple myeloma (180). Azacytidine might enhance sensitivity to lenalidomide and dexamethasone in patients with relapsed and/or refractory multiple myeloma. The efficacy of combined nivolumab and ibrutinib was previously assessed in patients with chronic lymphocytic leukemia (CLL) in a phase 2 clinical trial (181). Of 24 patients, 10 responded to this combined treatment, which corresponded to an overall response rate of 42%. Recently, fifty patients with advanced acral melanoma were enrolled in a phase 2 nonrandomized clinical study (182). Camrelizumab plus apatinib and temozolomide treatment contributed to a disease control rate of 88.0% in these patients. In addition, extensive clinical trials are planned or are currently underway to expand therapeutic avenues and improve patient outcomes, relevant examples of which are summarized in **Table 3**. Despite the encouraging clinical results, continual research efforts are warranted to reveal the mechanism by which PCD-targeted pharmaceuticals affect immunotherapy response in cancer patients.

**Table 2:** Summary of clinical trials testing combined programmed cell death-regulating drugs and immunotherapy in diverse cancer types.

Treatment	Route of administration	Cancer type	Type of study	Number of subjects	Outcome	Reference
NK cell therapy/trasuzumab	Intravenous	HER2-postive refractory breast and gastric cancers	Phase 1, open-label trial	22	Exhibit a good safety profile; result in clinically meaningful disease stabilization in six patients	(173)
CIK cell immunotherapy/sintilimab/carboplatin/pemetrexed; CIK cell immunotherapy/carboplatin/paclitaxel	Intravenous	Non-small-cell lung cancer	Single-center, open-label, phase 1b trial	34	Exhibit good tolerance and encouraging efficacy	(174)
Tiragolumab/atezolizumab	Intravenous	Non-small-cell lung cancer	Phase 2, international, multicenter, randomized, double-blind, placebo-controlled study	135	Improve objective response rate and progression-free survival	(175)
Durvalumab/carboplatin/paclitaxel; durvalumab/cisplatin/gemcitabine; durvalumab/pemetrexed/cisplatin; durvalumab/pemetrexed/carboplatin	Intravenous	Non-small-cell lung cancer	Phase 3, double-blind, placebo-controlled, multicenter, international trial	800	Prolong progression-free survival and overall survival; exhibit a tolerable safety profile	(176)
Pembrolizumab/gemcitabine; pembrolizumab/docetaxel; pembrolizumab/pemetrexed	Intravenous	Non-small-cell lung cancer	Single-arm, multicenter, phase 2 clinical trial	35	Prolong progression-free survival	(177)
Sitravatinib/tislelizumab	Oral/intravenous	Non-small-cell lung cancer	Open-label, multicenter, single-arm, non-randomized phase 1b clinical trial	115	Achieve a disease control rate of 88.5%	(178)
Paclitaxel/PD-(L)1 inhibitor	Intravenous	Non-small-cell lung cancer	Three-center, open-label, single-group, phase 2 study	29	Improve objective response rate	(179)
Azacitidine/lenalidomide/dexamethasone	Subcutaneous	Relapsed and/or refractory multiple myeloma	Phase 1b trial	42	Lead to an overall response rate of 22% and a clinical benefit response rate of 32%	(180)
Nivolumab/ibrutinib	Intravenous	Chronic lymphocytic leukemia	Phase 2 clinical trial	24	Achieve an overall response rate of 42%	(181)
Camrelizumab/apatinib/temozolomide	Intravenous/oral	Advanced acral melanoma	Single-arm, single-center, phase 2 nonrandomized clinical trial	50	Contribute to a disease control rate of 88.0%	(182)

**Table 3:** List of ongoing clinical trials testing combined programmed cell death-regulating drugs and immunotherapy in diverse cancer types.

Treatment	Cancer type	Type of study	Estimated enrollment	Status	Clinical trial identifier
Trastuzumab/fluoropyrimidine/platinum/pembrolizumab	HER2-positive esophageal squamous cell carcinoma	Phase 2 clinical trial	24	Recruiting	NCT05170256
Atezolizumab/carboplatin/nab-paclitaxel; pembrolizumab/platinum/pemetrexed	Lung adenocarcinoma	Open-label randomized, controlled, multicenter, phase 2 trial	136	Not yet recruiting	NCT05689671
Cemiplimab/platinum-doublet chemotherapy/pemetrexed/paclitaxel/fianlimab	Lung cancer	Phase 1 clinical trial	145	Recruiting	NCT03233139
SAR444881/pembrolizumab/carboplatin/pemetrexed	Advanced solid tumors (e.g., breast cancer, cervical cancer and colorectal cancer)	Phase 1 clinical trial; phase 2 clinical trial	456	Recruiting	NCT04717375
Tiragolumab/atezolizumab/pemetrexed/carboplatin; tiragolumab/atezolizumab/pemetrexed/cisplatin	Non-small cell lung cancer	Phase 2/3, randomized, double-blind, placebo-controlled study	540	Recruiting	NCT04619797
EOC202/paclitaxel	Hormone receptor-positive and HER2-negative advanced breast cancer	Phase 2 clinical trial	50	Not yet recruiting	NCT05322720
Abemaciclib/paclitaxel	Solid tumors	Open-label, multicenter phase 1b/2 study	30	Active, not recruiting	NCT04594005
Pembrolizumab/ibrutinib/rituximab	Primary central nervous system lymphoma	Phase 1b/2 clinical trial	37	Recruiting	NCT04421560
Ibrutinib/rituximab/lenalidomide	Recurrent/refractory primary or secondary central nervous system lymphoma	Phase 1b clinical trial	25	Active, not recruiting	NCT03703167
PCI-32765/rituximab/bendamustine hydrochloride	Relapsed diffuse large B-cell lymphoma, mantle cell lymphoma, indolent non-Hodgkin’s lymphoma	Phase 1, dose-escalation trial	48	Active, not recruiting	NCT01479842
Ibrutinib/cetuximab; Ibrutinib/nivolumab	Head and neck squamous cell carcinoma	Open-label, randomized, phase 2 trial	5	Active, not recruiting	NCT03646461
Ibrutinib/pembrolizumab	Melanoma	Phase 1 clinical trial	20	Active, not recruiting	NCT03021460
Camrelizumab/apatinib mesylate	High-risk gestational trophoblastic neoplasia	Open-label, phase 2 trial	73	Recruiting	NCT05139095
KN046/regorafenib/apatinib	Digestive system cancers	Phase 2 clinical trial	39	Recruiting	NCT06099821
CIK cell immunotherapy/apatinib	Advanced gastric cancer	Randomized, controlled, multicenter phase 2 clinical trial	80	Active, not recruiting	NCT02485015
Camrelizumab/apatinib mesylate	Advanced gastrointestinal cancer	Single-arm, open-label, phase 2 clinical trial	150	Recruiting	NCT05225844
Camrelizumab/apatinib	Triple-negative breast cancer	Open-label, multicenter, single-arm, investigator-initiated, phase 2 clinical trial	58	Recruiting	NCT05556200

## 5 Conclusions and future perspectives

It is increasingly acknowledged that multiple PCD pathways act under both physiological and pathological conditions. Due to its important role in cancer biology, PCD has become a hot spot in cancer research. Remarkably, various forms of PCD, such as apoptosis, autophagy-dependent cell death, pyroptosis and ferroptosis, are immunogenic and can therefore affect the immune system. Importantly, these PCD pathways exert a regulatory action on immune cells within the TME. The study of PCD in tumor immunity is a rapidly developing field. Accumulating evidence shows that PCD acts as a double-edged sword in cancer pathogenesis. PCD synergizes with host antitumor immune responses while contributing to cancer immunosuppression and immune evasion. The role of PCD in remodeling the TME is still poorly understood, and it was not until recently that the regulation and mechanisms of pyroptosis and ferroptosis were gradually recognized. Therefore, the impact of these newly characterized forms of PCD on tumor immunity deserves special attention. The traditional view holds that apoptotic cell death is an immunologically “silent” process and cannot trigger inflammation. However, emerging evidence suggests that certain stimuli can elicit an immunogenic subtype of apoptosis in cancer (184). Investigations of ICD, especially immunogenic apoptosis, have just begun. Accordingly, considerable research efforts are needed to gain a thorough comprehension of the broad communication between PCD and antitumor immunity. This interaction pattern may even vary in distinct tumor types or in different contexts. It will be equally important to determine how cancer cells and diverse cells within the TME interact with each other to activate or block tumor immunity. An in-depth investigation of the complex TME may help to elucidate the mechanisms underlying PCD-mediated immune regulation. Cancer cell populations can simultaneously undergo various types of PCD. Different forms of PCD may act in synergy or in opposition at the same tumor site. Certain cell death pathways can induce or restrict other death mechanisms. It is thus necessary to adequately decipher the sophisticated regulatory network behind distinct forms of PCD in the TME and define which PCD type plays a dominant role in different contexts. Moreover, factors that determine the ultimate effect of various types of PCD on cancer progression and tumor immunity must be carefully identified.

According to the available evidence from both preclinical and clinical studies, PCD can affect the efficacy of immunotherapy through crosstalk with immune cells infiltrating the TME. Manipulation of cell death pathways seems to be an attractive strategy to enhance the treatment advantages of cancer immunotherapy. A variety of antitumor agents can affect the immunotherapy response by initiating cell death programs in cancer. The exact mechanisms responsible for the synergistic actions of chemotherapeutic agents and ICB require further investigation. Noncancerous cells, including normal cells and immune cells, may also die upon recognition of signals (e.g., DAMPs) released from dying cancer cells. It remains uncertain whether chemotherapy-induced PCD is beneficial for cancer patients in the long term. The side effects of chemotherapy in combination with immunotherapy must be completely examined. The screening and discovery of tumoricidal drugs that specifically act on cancer cells with minimal adverse effects on normal cells are urgently needed. Tumor-targeted delivery systems, such as exosomes and polymeric nanoparticles, seem to offer an effective way to deliver therapeutic agents to cancer cells (185). The development of safe and efficient delivery vehicles may help to balance therapeutic goals and potential side effects. The administration sequence and timing of various drugs in combination therapies need to be determined. In addition, further clinical studies are needed to investigate the efficacy, safety and mechanisms of antineoplastic drugs combined with immunotherapy in large cohorts of cancer patients. Taken together, gaining better insight into PCD involvement in carcinogenesis and tumor immunity will finally accelerate the translation of scientific discoveries into new ways to treat cancer.

## Author contributions

MW: Conceptualization, Funding acquisition, Supervision, Visualization, Writing – original draft. FY: Investigation, Resources, Writing – review & editing. YZ: Writing – review & editing, Resources. PL: Conceptualization, Supervision, Writing – review & editing.
